# Type three secretion system-mediated escape of *Burkholderia pseudomallei* into the host cytosol is critical for the activation of NFκB

**DOI:** 10.1186/1471-2180-14-115

**Published:** 2014-05-06

**Authors:** Boon Eng Teh, Christopher Todd French, Yahua Chen, Isabelle Gek Joo Chen, Ting-Hsiang Wu, Enrico Sagullo, Pei-Yu Chiou, Michael A Teitell, Jeff F Miller, Yunn-Hwen Gan

**Affiliations:** 1 Department of Biochemistry, Yong Loo Lin School of Medicine, National University of Singapore, Singapore 117597, Singapore; 2 Department of Microbiology, Immunology and Molecular Genetics, Los Angeles, CA 90095, USA; 3 Department of Pathology and Laboratory Medicine, Los Angeles, CA 90095, USA; 4 Department of Mechanical and Aerospace Engineering, Los Angeles, CA 90095, USA; 5 California NanoSystems Institute, Los Angeles, CA 90095, USA; 6 Broad Stem Cell Research Center, Los Angeles, CA 90095, USA; 7 Jonsson Comprehensive Cancer Center, The University of California Los Angeles, Los Angeles, CA 90095, USA; 8 Molecular Biology Institute, The University of California Los Angeles, Los Angeles, CA 90095, USA; 9 Immunology Program, National University of Singapore, Singapore 117597, Singapore

## Abstract

**Background:**

*Burkholderia pseudomallei* is the causative agent of melioidosis, a potentially fatal disease endemic in Southeast Asia and Northern Australia. This Gram-negative pathogen possesses numerous virulence factors including three “injection type” type three secretion systems (T3SSs). *B. pseudomallei* has been shown to activate NFκB in HEK293T cells in a Toll-like receptor and MyD88 independent manner that requires T3SS gene cluster 3 (T3SS3 or T3SS_Bsa_). However, the mechanism of how T3SS3 contributes to NFκB activation is unknown.

**Results:**

Known T3SS3 effectors are not responsible for NFκB activation. Furthermore, T3SS3-null mutants are able to activate NFκB almost to the same extent as wildtype bacteria at late time points of infection, corresponding to delayed escape into the cytosol. NFκB activation also occurs when bacteria are delivered directly into the cytosol by photothermal nanoblade injection.

**Conclusions:**

T3SS3 does not directly activate NFκB but facilitates bacterial escape into the cytosol where the host is able to sense the presence of the pathogen through cytosolic sensors leading to NFκB activation.

## Background

*Burkholderia pseudomallei*, the causative agent of melioidosis, is a highly versatile Gram-negative bacterium capable of invading epithelial cells [1] as well as surviving in macrophages [2]. Common routes of entry for *B. pseudomallei* are via cutaneous inoculation, inhalation, or ingestion. Melioidosis is endemic in Southeast Asia, Northern Australia and other tropical regions [3], and clinical outcome is relatively dependent on the size of the inoculum and the existence of predisposing risk factors [4]. *B. pseudomallei* possesses an extensive arsenal of recognized virulence determinants, including three “injection type” type III secretion systems (T3SSs) and six type VI secretion systems (T6SSs). T3SSs are present in many Gram-negative pathogens and translocate “effector” proteins into eukaryotic host cells to alter their cellular response. In *B. pseudomallei*, only T3SS3 has been implicated in animal pathogenesis [5,6], while T3SS1 and −2 are predicted to mediate interactions with plants [7]. T3SS3 has also been shown to be important for bacterial escape from phagosomes or endosomes into the host cytosol [8,9] and caspase 1-induced pyroptosis [10].

Since T3SS is a virulence determinant utilized by a variety of Gram-negative species, mammalian hosts have evolved sensors to detect the presence of T3SSs during pathogenesis. In macrophages, the T3SS of *Salmonella typhimurium*, *Shigella flexneri, B. pseudomallei, Pseudomonas aeruginosa,* enterohemorrhagic and enteropathogenic *E. coli* trigger a proinflammatory response mediated by the NLRC4 inflammasome and subsequent activation of caspase 1 [11]. In *Yersinia*, it is unclear whether caspase 1 activation is triggered by the translocon pore or via unknown T3SS-related factors [12]. In addition to detection by the inflammasome machinery, *Yersinia*[13] and *Salmonella*[14] can be detected by NFκB in a Toll-like receptor (TLR) and MyD88 independent manner that is reliant on T3SS, revealing another possible mechanism whereby T3SS can be detected by host epithelial cells which lack inflammasome machinery. Using human embryonic kidney cells (HEK293T), which are epithelial cells that lack TLR 2, 4 and 9 expression but expresses low levels of TLR5 and 7 [15,16], we have previously shown that *B. pseudomallei* stimulates NFκB independently of TLRs and MyD88, leading to the production of IL-8. NFκB activation required bacterial internalization and a functional T3SS3 [17]. However, it is unclear whether NFκB activation is triggered by T3SS3 effector proteins, by components of the T3SS secretion apparatus itself, or indirectly via additional T3SS3-mediated processes.

Our goal is to determine how T3SS3 contributes to NFκB activation in the absence of TLR, MyD88 and inflammasome signalling using HEK293T epithelial cells as a model system. We show that T3SS3-mediated endosome escape is required for NFκB activation and occurs independently of known T3SS3 effector proteins. Using a photothermal nanoblade to directly place bacteria into the cytoplasm, we show that cytosolic localization is sufficient to activate NFκB. Thus, *B. pseudomallei* T3SS3 is not directly detected by the host NFκB pathway but is instead responsible for bacterial escape from vacuolar compartments subsequently leading to the activation of cytosolic sensors.

## Results

### TLR-independent NFκB activation by B. pseudomallei is dependent on the activity of T3SS3 but not known T3SS3 effector proteins

**Figure 1:**
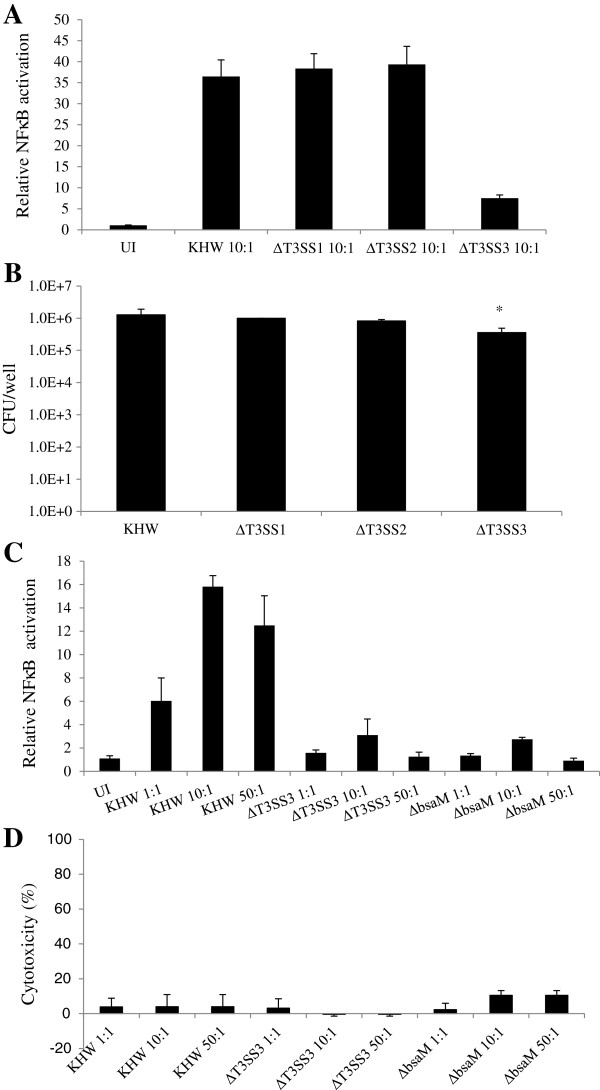
**TLR independent NFκB activation by *B. pseudomallei *requires T3SS3. A)** HEK293T cells were transfected with pNFκB-SEAP for 24 hr. The transfected cells were infected with wildtype KHW and mutants at MOI of 10:1 for 6 hr. Supernatants were collected for SEAP assay. **B)** HEK293T cells were infected with respective strains for 6 hr. Cells were lysed and plated for intracellular bacterial count. **C)** HEK293T cells were transfected with pNFκB-SEAP for 24 hr. The transfected cells were infected with wildtype KHW and mutants at indicated MOI for 6 hr. Supernatants were collected for SEAP assay. **D)** HEK293T cells were infected with respective strains for 6 hr. Supernatants were collected for lactate dehydrogenase (LDH) assay. Asterisks * and ** indicate significant differences of *p* < 0.05 and *p* < 0.01 between *B. pseudomallei* wildtype and mutant strains respectively.

We had previously shown that activation of NFκB in HEK293T cells by *B. pseudomallei* was not dependent on host TLR and MyD88 signalling but required a functional bacterial T3SS3 [17]. Here, we first investigate whether *B. pseudomallei* T3SS1 and T3SS2 contribute to NFκB activation, or if it is a specific consequence of T3SS3 activity. Derivatives of *B. pseudomallei* strain KHW containing deletions of the entire T3SS3, T3SS2 or T3SS1 gene clusters were constructed by allelic exchange. HEK293T cells that were transiently transfected with the NFκB-SEAP (secreted embryonic alkaline phosphatase) reporter system were infected with wildtype KHW or mutant strain, and assayed for NFκB activation 6 hr. later. As shown in Figure 1A, infection with the ΔT3SS3 strain showed reduced NFκB activation in contrast to the ΔT3SS1 and ΔT3SS2 mutant derivatives, which led to robust activation comparable to wildtype bacteria. As the ΔT3SS3 mutant was unable to replicate as well as wildtype KHW and the other mutants (Figure 1B), the lack of NFκB activation could be due to lower bacterial numbers. Furthermore, it is known that complete deletion of T3SS3 also inactivates T6SS1 due to removal of T6SS1 regulatory loci located in the T3SS3 gene cluster [18]. To determine whether NFκB activation is dependent on the activity of T3SS3 or T6SS1, a strain containing an in-frame deletion in *bsaM*, which encodes an inner membrane ring component of T3SS3 that is essential for function, was assayed in parallel [19]. The Δ*bsaM* mutation does not affect T6SS regulatory loci that are present in the T3SS3 gene cluster. The results in Figure 1C demonstrate that infection with the Δ*bsaM* and the ΔT3SS3 mutants leads to equivalently low levels of NFκB activation compared to wildtype KHW, even at high multiplicity of infection (MOI). All subsequent experiments were then performed with the Δ*bsaM* mutant instead of the ΔT3SS3 mutant. The amount of bacterial-induced cellular cytotoxicity was very low (10% or less) and comparable across all strains and MOIs (Figure 1D), showing that difference in NFκB activation is not due to differing levels of cell death. The lack of increase in NFκB activation at MOI of 50:1 could be due to NFκB suppression mediated by the presence of TssM in the strains, as we had previously reported [20].

**Figure 2:**
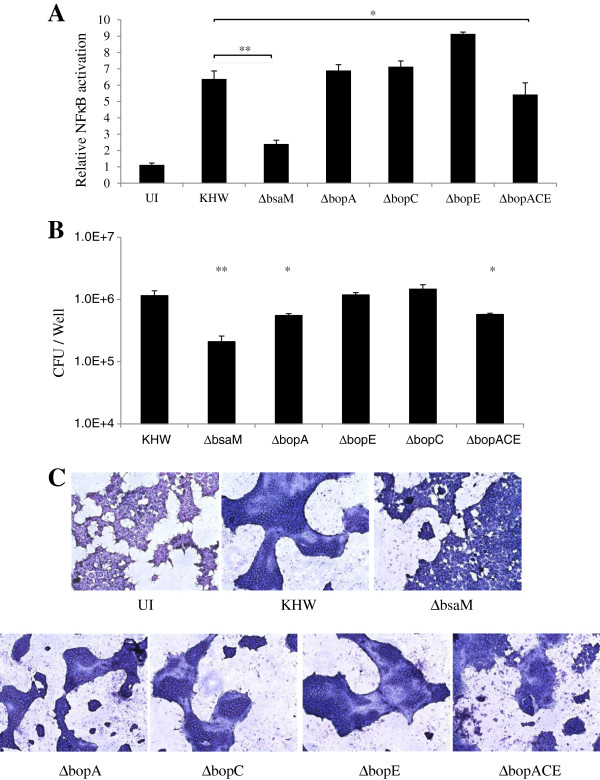
**Deletion of T3SS3 effector genes had little effect on TLR independent NFκB activation by *B. pseudomallei*. A)** HEK293T cells were transfected with pNFκB-SEAP for 24 hr. The transfected cells were infected with wildtype KHW and mutants at MOI of 10:1 for 6 hr. Supernatants were collected for SEAP assay. **B)** HEK293T cells were infected with respective strains for 6 hr. Cells were lysed and plated for intracellular bacterial count. **C)** HEK293T cells were infected with respective strains for 12 hr. The infected cells were fixed, stained with Giemsa and visualized under 10x magnification on a light microscope. Asterisks * and ** indicate significant differences of *p* < 0.05 and *p* < 0.01 between *B. pseudomallei* wildtype and mutant strains respectively.

The role of T3SS is to translocate effector proteins into the eukaryotic cell interior. Unlike the T3SSs of some other pathogenic species such as *Salmonella* and *Shigella*, *B. pseudomallei* T3SS3 possesses only three known effectors; BopA [21], BopC [22], and BopE [23]. When cells were infected with Δ*bopA*, Δ*bopC* or Δ*bopE* strains and NFκB activation was measured at 6 hr. after infection, no significant difference was observed compared to wildtype KHW. In the case of the ∆*bsaM* mutant, activation was minimal as expected, whereas the ∆*bopACE* triple effector mutant showed a slight reduction in NFκB activation (5.4 fold) compared to wildtype bacteria (6.4 fold) (Figure 2A). Moreover, the ∆*bsaM* strain exhibited an approximately 5.5-fold reduction in the numbers of intracellular bacteria compared to wildtype bacteria at the same 6 hr. time point, while Δ*bopACE* was only slightly (2 fold) reduced (Figure 2B), corresponding with their respective abilities to activate NFκB shown in Figure 2A. Thus, lower NFκB activation is likely due to lower replication rates of the ∆*bsaM* and Δ*bopACE* mutants, and does not seem to be contributed by the known T3SS3 effectors.

T3SS3 does not facilitate invasion of bacteria into cells but rather promotes their subsequent escape from endocytic vesicles [24]. Therefore, defective endosome escape by mutants may provide an explanation for their reduced replication and inability to activate NFκB. Thus, we examined whether the ability of these mutants to activate NFκB correlate with their ability to escape from the endosome. The formation of multinucleated giant cells (MNGC) at 10–12 hr. following infection was utilized as a measure of endosome escape, since it requires the activity of T6SS1 and only occurs if bacteria have escaped from endocytic vesicles into the cytosol [18,24]. We examined the formation of MNGC at 12 hr. post infection of the single and triple effector mutants in comparison with wildtype KHW and the escape-deficient Δ*bsaM* (Figure 2C). All strains could induce MNGC at this time-point except for Δ*bsaM*, indicating that the ability to activate NFκB correlates with the ability to escape. Δ*bopACE* formed less MNGCs compared to the rest, likely reflecting its lower replication ability.

**Figure 3:**
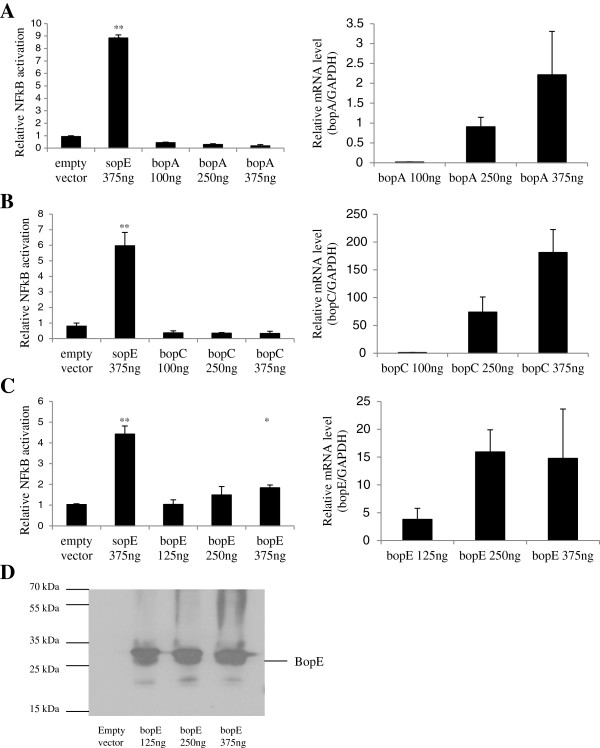
**TLR independent NFκB activation by *B. pseudomallei *is not dependent on T3SS3 effectors.** HEK293T cells were cotransfected with pNFκB-SEAP and mammalian expression vectors encoding genes for BopA **(A)** BopC **(B)** and BopE **(C)** for 24 hr. Supernatants were collected for SEAP assay (left panels). Total RNA was isolated for measuring of expression of effector genes (right panels) by real-time PCR. **D)** Cells transfected with BopE plasmid were lysed and analysed by Western blot with anti-BopE antibody. SopE was used as a positive control. Asterisks * and ** indicate significant differences of *p* < 0.05 and *p* < 0.01 between empty vector and plasmid expressing T3SS effector gene respectively.

Another possibility is that the Δ*bsaM* and Δ*bopACE* strains are defective in the secretion of T3SS3 effector proteins, which could be responsible for activating NFκB as has been reported for the T3SS effector proteins SopE and SipA from *Salmonella*[25]. This is unlikely given that our single effector mutants could still activate NFκB as well as wildtype bacteria. To confirm, BopA (Figure 3A), BopC (Figure 3B) or BopE (Figure 3C) were ectopically expressed in increasing plasmid concentrations in HEK293T cells. None of the *Burkholderia* effectors were able to activate NFκB significantly above background levels with the exception of BopE (Figure 3C), a homolog of *Salmonella* SopE, which showed only a slight activation. In contrast, expression of *Salmonella* SopE led to robust activation. We verified that the proteins were indeed expressed at the mRNA level (Figure 3A-C) as well as at the protein level for BopE (Figure 3D). It is therefore doubtful that individual T3SS3 effectors are responsible for NFκB activation in HEK293T cells, but that activation likely depends on T3SS3-mediated escape from endocytic vesicles following invasion.

### T3SS3 mutants activate NFκB when they gain access to the host cytosol

**Figure 4:**
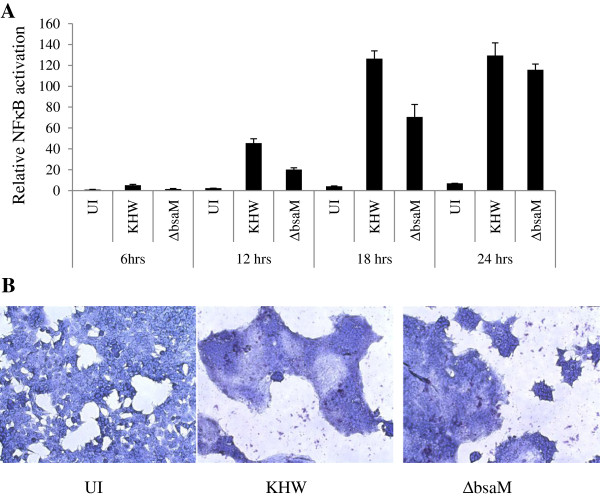
**T3SS3 mutants activate NFκB at late time-points corresponding to escape into cytosol. A)** HEK293T cells were transfected with pNFκB-SEAP for 24 hr. The transfected cells were infected with wildtype KHW and Δ*bsaM* at MOI of 10:1. Supernatants were collected at respective time points for SEAP assay. **B)** HEK293T cells were infected with wildtype KHW and Δ*bsaM* at MOI of 10:1 for 18 hr. The infected cells were fixed, stained with Giemsa and visualized under 10x magnification on a light microscope.

It is known that T3SS3 facilitates escape from phagosomal or endosomal compartments into the host cell cytosol [8,24], although *B. pseudomallei* T3SS3 mutants have been observed to exhibit delayed escape via an unidentified mechanism [8]. A time-course of NFκB activation shows that the T3SS3 mutant ∆*bsaM* was unable to activate NFκB at 6 hr. after infection, although it was increasingly able to do so when the incubation was extended to 24 hr. (Figure 4A), where levels became comparable to infection with wildtype KHW. In Figure 2C, we had shown that ∆*bsaM* mutant was unable to form MNGCs at 12 hr., corresponding to their inability to activate NFκB at early time-points. By 18 hr., both wildtype KHW and ∆*bsaM* mutant induced the formation of MNGCs (Figure 4B). On the basis of these observations, we hypothesized that T3SS-independent escape from endosomes is responsible for NFκB activation by the ∆*bsaM* mutant at later time points, and the critical event required for NFκB activation is bacterial entry into the cytosol.

If NFκB activation at early time points results from rapid escape from the endosome, then direct placement of bacteria into the cytosol should obviate the need for T3SS-mediated escape. This was tested using a photothermal nanoblade, which allows us to bypass the need for invasion and endosome escape altogether [24,26]. The photothermal nanoblade utilizes a 6 ns pulse from a 540 nm laser to excite a titanium coating on glass micropipettes that are brought into contact with mammalian cell membranes. Rapid heating results in the formation of a vapour “nanobubble”, creating a local, transient delivery portal in the membrane bilayer through which cargo can be introduced. The advantages of photothermal nanoblade compared to traditional microinjection are that variably-sized particles – from molecules to bacteria - can be efficiently delivered into a wide range of cell types, and cell viability is maintained since physical puncturing does not occur.

**Figure 5:**
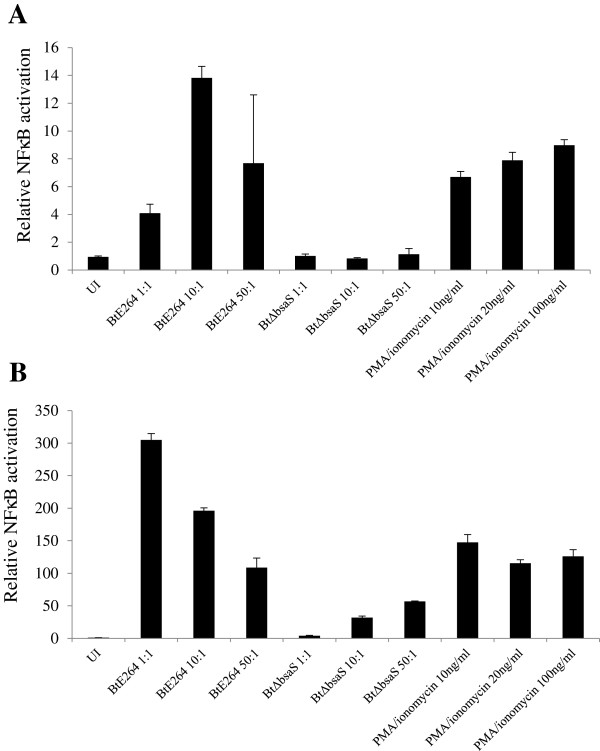
***B. thailandensis *T3SS3 mutants activate NFκB.** NFκB/293/GFP-Luc cells were infected with wildtype *B. thailandensis* (E264), *B. thailandensis* ∆*bsaS* mutant or stimulated with PMA and ionomycin for 6 hr **(A)** and 24 hr **(B)**. Cells were lysed and assayed for luciferase activity.

**Figure 6:**
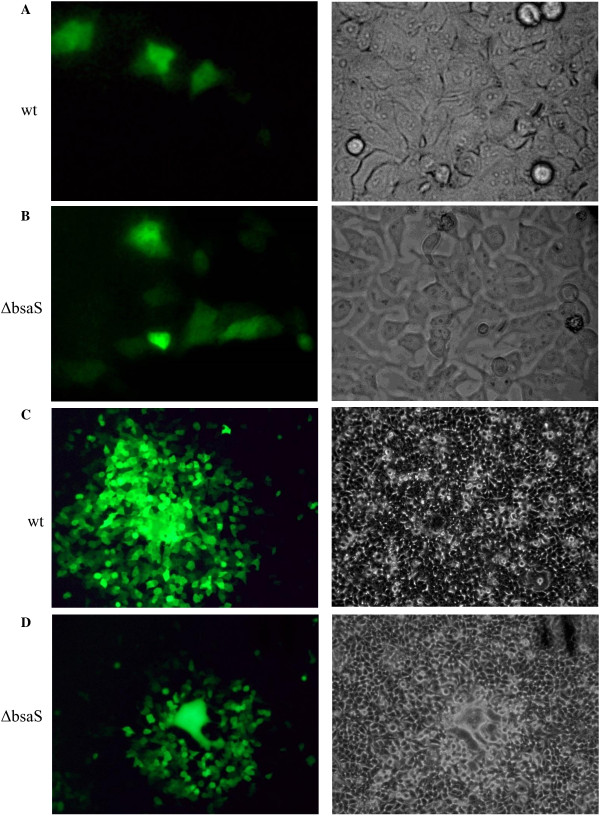
**Direct delivery of T3SS3 mutant into the cytosol activates NFκB.** NFκB/293/GFP-Luc cells were injected with wildtype *B. thailandensis* (E264) **(A)** or *B. thailandensis ΔbsaS***(B)** for 6 hr or 24 hr **(C, D)**. The infected cells were observed under the fluorescence microscope (40x magnification for 6 hr and 10x magnification for 24 hr) to monitor for GFP production as an indication of NFκB activation. The right panel represents an image taken under bright field microscope illumination whereas the left panel shows an image taken under fluorescence illumination.

*B. thailandensis* was used for these experiments since the instrument is not adapted for use in a BSL-3 environment. *B. thailandensis* encodes a T3SS apparatus (T3SS_Bsa_) that is highly homologous to *B. pseudomallei* T3SS3 and functions in an analogous manner [24,27]. Its intracellular growth and intercellular spread characteristics are comparable to *B. pseudomallei*, making it a useful surrogate for studying the *Burkholderia* intracellular life cycle. We first established that NFκB activation is dependent on *B. thailandensis* T3SS_Bsa_, as the T3SS_Bsa_ mutant ∆*bsaS*[24] did not markedly activate NFκB at 6 hr. after infection at an MOI of 10:1 (Figure 5A), but did so at 24 hr. using the same MOI (Figure 5B), similar to what was seen with *B. pseudomallei* (Figure 4A). *bsaS* encodes the ATPase for T3SS_Bsa_, and *B. pseudomallei* and *B. thailandensis* ∆*bsaS* derivatives have been shown to be deficient in T3SS_Bsa_ function, including lower intracellular replication [24]. PMA and ionomycin treatment served as positive controls for the photothermal nanoblade experiments, and NFκB /293/GFP-Luc cells were used so that NFκB activity could be measured by luciferase activity as well as GFP fluorescence. We were struck by the finding that 6 hr. after photothermal nanoblade delivery of bacteria into the host cell cytosol, both wildtype bacteria (Figure 6A) and the ∆*bsaS* mutant showed comparable GFP fluorescence and hence, NFκB activation (Figure 6B). Uninfected cells did not produce detectable GFP fluorescence (data not shown). Similarly, both the wildtype and ∆*bsaS* mutant bacteria activated NFκB extensively at 24 hr. following nanoblade delivery (Figure 6C, D). Taken together, these results demonstrate that T3SS_Bsa_ mutants are able to activate NFκB effectively at early time-points if the need to escape from vacuolar compartments is bypassed by direct delivery of bacteria into the cytosol.

### B. pseudomallei stimulates activation of endogenous NFκB in HEK293T cells

**Figure 7:**
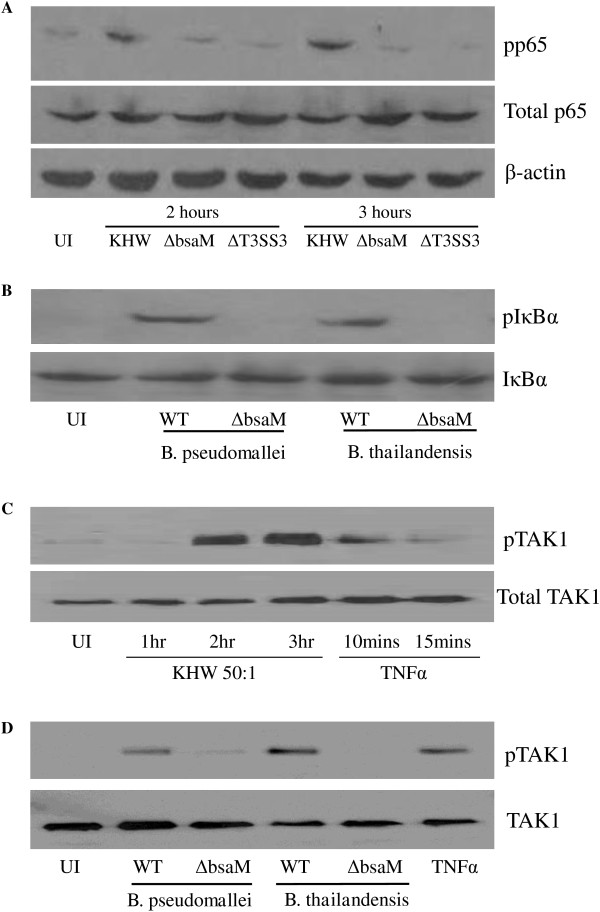
***B. pseudomallei *wildtype but not the T3SS3 mutant induces p65, IκBα and TAK1 phosphorylation. A)** HEK293T cells were infected with *B. pseudomallei* strains at MOI 50:1. Cells were lysed at 2 and 3 hr and analyzed by Western blot with anti-phospho-p65, anti-p65 and anti-β-actin antibodies. **B)** HEK293T cells were infected with *B. pseudomallei* and *B. thailandensis* strains at MOI 50:1. Cells were lysed at 2 hr and analyzed by Western blot with anti-phospho-IκBα and anti-IκBα antibodies. **C)** HEK293T cells infected with KHW at MOI 50:1. Cells were lysed at 1, 2 and 3 hr. Lysates were immunoprecipitated with anti-TAK1 antibody and immunoblotted with phospho-TAK1 antibody. The TNFα stimulated cells were used as a positive control. **D)** HEK293T cells were infected with *B. pseudomallei* and *B. thailandensis* strains at MOI 50:1. Cells were lysed at 2 hr. Lysates were immunoprecipitated with anti-TAK1 antibody and immunoblotted with phospho-TAK1 antibody. The TNFα stimulated cells were used as a positive control.

As previous experiments involved activation of an NFκB reporter, we wanted to measure endogenous levels of NFκB activity in HEK293T cells infected with *B. pseudomallei*. To this end, we measured the phosphorylation of key NFκB signalling intermediates beginning with the most downstream signalling molecule in the pathway, the NFκB p65 subunit. Infection of cells with wildtype bacteria, but not ΔT3SS3 or Δ*bsaM* mutants, led to a pronounced increase in phosphorylated p65, whereas total p65 remained constant at 2 hr. and 3 hr. post infection (Figure 7A). Phosphorylation of the central IκBα was also seen following infection with wildtype bacteria, but not with *B. pseudomallei* and *B. thailandensis* ∆*bsaM* mutants (Figure 7B). A key signalling intermediate in the NFκB activation pathway is TAK1, which lies upstream of the IKK complex and is triggered by various stimuli such as TNFα, IL-1β, TLRs, TGFβ and DNA damage [28]. We found that *B. pseudomallei* infection resulted in a time-dependent increase in phosphorylated TAK1 (Figure 7C), which was greatly reduced following infection with *B. pseudomallei* and *B. thailandensis* ∆*bsaM* mutants (Figure 7D). Thus, these experiments show that infection with wildtype bacteria, but not T3SS3-defective mutants, leads to endogenous NFκB activation accompanied by activation of TAK1, in agreement with our previous data with the NFκB reporter assays.

## Discussion

Several Gram-negative bacterial pathogens capable of infecting epithelial cells possess secretion systems such as T3SS or T4SS that modulate NFκB signalling. In *Legionella pneumophila*, NFκB activation was shown to occur via a TLR dependent pathway, as well as a TLR-independent pathway that requires the Icm/Dot translocation system [29-32]. Recently, a Icm/Dot substrate LnaB has been identified to be responsible for TLR-independent activation of NFκB with activation of RIP2 (common downstream intermediate of NOD1 and NOD2) in HEK293T cells [33]. Another T4SS secreted effector, LegK1, activates NFκB directly by phosphorylating NFκB inhibitor IκBα, leading to downstream activation independent of host PRRs [34].

Intestinal pathogens such as *Salmonella* and *Shigella* have been shown to activate NFκB in intestinal epithelial cells in a TLR independent manner. For example, *Shigella flexneri* invades and activates NOD1, which senses bacterial peptidoglycan, leading to IL-8 production [35]. In *Salmonella*, the T3SS effector SopE activates NFκB [36] by engaging small Rho GTPases CDC42 and Rac1, which in turn trigger NOD1 and RIP2 activation of NFκB [25]. Another *Salmonella* T3SS effector protein SipA was also found to activate NFκB via NOD1/NOD2 signalling pathway that proceeds through RIP2 [37]. In contrast, it cannot be definitively determined in *Yersinia* whether the T3SS cargo or translocon pore is responsible for activating NFκB [13].

In this study, we have shown that *B. pseudomallei* and *B. thailandensis* T3SS3 do not directly activate NFκB in any significant way in HEK293T epithelial cells. T3SS3 is necessary for efficient escape of bacteria from endosomal/phagosomal compartments into the cytosol at early time-points, although some escape may occur with low efficiency at later time-points independently of T3SS3 [8]. Although the direct delivery of T3SS3 mutants was done only with *B. thailandensis*, the time course of MNGC formation and NFκB activation of *B. pseudomallei* ∆*bsaM* mutants, and the similarity in various parameters between the two species in our experiments as well as what has been reported in the literature [23,26] would support our conclusion. In contrast to what has been found for *Salmonella*, known T3SS3 effectors are not essential for NFκB activation by *Burkholderia*. This is supported by several lines of evidence: T3SS mutant bacteria exhibit delayed but significant NFκB activation at later time-points, corresponding to their escape into the cytosol; overexpressed T3SS3 effectors do not activate NFκB; and direct delivery of bacteria into the cytosol via nanoblade injection obviates the need for T3SS3 in NFκB activation even at early time-points. Thus, the key event triggering NFκB activation is the presence of *Burkholderia* in the cytoplasm. We have not completely ruled out the possibility that unknown T3SS3 effectors secreted by other T3SSs in the absence of T3SS3 may partly be responsible for the NFκB activation we see, but even if this is true, it likely plays a minor role as the activation would not have depended so much on the cytosolic presence of the bacteria. The requirement for cytosolic presence of the pathogen likely reflects the host’s reliance on cytosolic sensors to detect generic pathogen associated molecular patterns (PAMPs) rather than the specific recognition of T3SS- or T4SS-associated proteins as seen for pathogens that depend on survival within a vacuolar compartment such as *Salmonella* and *Legionella*. However, we cannot rule out the possibility that the cytosolic presence of bacteria expose T3SS3 structural components to activate NFκB.

The detection of endogenous TAK1 activation in HEK293T cells following infection with wildtype, but not T3SS3 mutants, suggests the activation of the intracellular pattern recognition receptors (PRRs) NOD1 and NOD2, both of which signal through TAK1. *B. pseudomallei* is reportedly able to signal through NOD2 in RAW264.7 macrophages to upregulate suppressor of cytokine signalling 3 (SOCS3) although it does not result in similar upregulation of the proinflammatory cytokines TNFα, IL-1β and IL-6 which depend on activation of NFκB [38]. Recently, it is reported that NOD2 plays a minor role in murine melioidosis and a human genetic polymorphism in NOD2 region is associated with melioidosis [39]. It is possible that NOD1 and NOD2, which sense bacterial peptidoglycan derivatives IE-DAP and muramyl dipeptide respectively, may be the major cytosolic sensors responsible for NFκB activation.

## Conclusions

Use of the HEK293T cells has allowed us to determine how *Burkholderia* T3SS3 contributes to NFκB activation in the absence of TLR and MyD88 signalling. We were able to discern that activation of NFκB does not occur as a direct consequence of *Burkholderia* T3SS3 secretion of effectors, but rather through cytosolic sensors that respond to the presence of bacteria in the cytosol following T3SS3-mediated escape from endocytic vesicles. Our study serves as a model for future work to identify the cytosolic sensors and the conditions leading to NFκB activation. It is possible that NFκB is not triggered efficiently by surface or endosomal PRRs, whereupon cytosolic sensors become important in establishing recognition of bacterial pathogens and eventual protection. Alternatively, the activation of these cytosolic sensors may lead to a different gene expression program that provides a regulatory function distinct from the TLR response.

## Methods

### Cell-lines and bacterial strains

Human embryonic kidney HEK293T (ATCC CRL-11268) cells were cultured in Dulbecco’s modified Eagle medium (Sigma-Aldrich) with 10% heat-inactivated fetal bovine serum (Life Technologies), 1X penicillin/streptomycin (Life Technologies) and 2 mM L-glutamine (Life Technologies) at 37°C with humidified atmosphere with 5% CO_2_. NFκB/293/GFP-Luc cell line was purchased from System Biosciences and cultured in the same medium as HEK293T cells. Bacterial strains used are listed in Table 1.

**Table 1:** List of bacterial strains used in this study

**Strain**	**Relevant characteristic(s)**^***a***^	**Source or reference**
*B. pseudomallei*		
KHW	*B. pseudomallei* wildtype strain	[40]
ΔT3SS1	T3SS1 cluster was replaced with tetracycline resistance gene, Tc^r^	[40]
ΔT3SS2	T3SS2 cluster was replaced with tetracycline resistance gene, Tc^r^	[40]
ΔT3SS3	T3SS3 cluster was replaced with zeocin gene, Zeo^r^	[40]
Δ*bsaM*	*bsaM* orf was deleted	This study
Δ*bopA*	*bopA* orf was replaced with zeocin resistance gene, Zeo^r^	This study
Δ*bopC*	*bopC* orf was replaced with zeocin resistance gene, Zeo^r^	This study
Δ*bopE*	*bopE* orf was deleted	This study
Δ*bopACE*	*bopA* and *bopE* orfs were deleted, *bopC* orf was replaced with zeocin resistance gene, Zeo^r^	This study
*B. thailandensis*		
E264	*B. thailandensis* wildtype strain	[41]
Δ*bsaS*	*bsaS* orf was deleted	[24]
Δ*bsaM*	*bsaM* orf was deleted	This study

^*a*^*Abbreviations*: Tc^r^, tetracycline resistant, Zeo^r^, zeocin resistant.

### Bacterial mutant construction

All plasmids used for mutant construction are listed in Table 2. *B. pseudomallei* and *B. thailandensis* gene deletions were generated by allelic exchange. Approximately 1 kb fragments upstream and downstream of the target gene were amplified from genomic DNA and cloned into pK18mobsacB vector [42] simultaneously using In-Fusion PCR cloning kit (Clontech). A zeocin resistance cassette from pUC18T-mini-Tn7T-Zeo-lox [43] was inserted between the gene fragments for some of the constructions. The plasmids were introduced into *B. pseudomallei* and *B. thailandensis* strains by conjugation. Homologous recombination was then selected for by growing bacteria in LB + 15% sucrose to counter select the *sacB* gene in the pK18mobsacB plasmid backbone. Successful double cross-over clones were screened by colony PCR from kanamycin sensitive colonies. Primers used for mutant construction are listed in Table 3.

**Table 2:** Plasmids used for cellular transfection in the study

**Plasmid**	**Relevant characteristic(s)**^***a***^	**Source or reference**
pK18mobsacB	Conjugative, suicide vector containing *sacB* gene, Km^r^	[42]
pUC18T-mini-Tn7T-Zeo-lox	Source of zeocin resistance gene, Ap^r^, Zeo^r^	[43]
pNFκB-SEAP	Reporter vector containing NFκB enhancer element fused to SEAP gene, Amp^R^	BD Clontech
pcDNA3.1/V5-His TOPO	Expression Vector, Amp^R^	Life Technologies
pCMV-FLAG-MAT-Tag-1	Expression Vector, Amp^R^	Sigma
pcDNA-bopA	*bopA* gene cloned into pcDNA3.1/V5-His TOPO by TA-cloning, Amp^R^	This study
pCMV-bopC	*bopC* gene cloned into pCMV-FLAG-MAT-Tag-1, Amp^R^	This study
pRK5myc-BopE	*bopE* gene cloned into pRK5*myc*, Amp^R^	[23]
pRK5myc-SopE	*sopE* gene cloned into pRK5*myc*, Amp^R^	[23]

^*a*^*Abbreviations*: Amp^R^, ampicillin resistant.

**Table 3:** List of primers for this study

**Primer name**	**Sequences (5′-3′)**
Mutant construction	
BsaM up for	AAGCTTCACGCGACGCGATTTTGAATTG
BsaM up rev	AAGCTTGCTCGCCGACGCAGAAAAATA
BsaM dn for	GAATTCAAGCTTGATCACGCGTCCTGGTATTT
BsaM dn rev	TTGGATCCAAGCGAGACGTAGATGCTG
BopA up for	CCAAGCTTGCATGCCTGCAGGTCTTGCTCTCGGTTGAAGG
BopA up rev	GAGGATCCCCGGGTATGCATCGACATTGATCATCC
BopA dn for	TACCCGGGGATCCTCGCATGAAGAACGCATGAAGA
BopA dn rev	CCATGATTACGAATTCGATTCTTGTTGCTCCGATGC
BopC up for	CCATGATTACGAATTCCCCGACCAGTTGAAGATGTC
BopC up rev	GAGGATCCCCGGGTAGAACCAATGCCTAGCCTCAC
BopC dn for	TACCCGGGGATCCTCCTGGGGTCGGTTTACATACG
BopC dn rev	CCAAGCTTGCATGCCTGCAGGAGCATCGCGAATACGAACT
BopE up for	CGGTACCCGGGGATCCAACAACCGCTCCTTCATCC
BopE up rev	TCATGTCTTGCTCTCGGTTG
BopE dn for	GAGAGCAAGACATGAGACGCTCGAAGCCACATAC
BopE dn rev	GGCCAGTGCCAAGCTTGTATTACGAGTCGGGGCTGA
Bt BsaM up for	GGCCAGTGCCAAGCTTTTTCCAGAAAAGCGAGCAAT
Bt BsaM up rev	GGCGATAAATGGCCTGATTA
Bt BsaM dn for	AGGCCATTTATCGCCGATCACGCGTCCTGGTATTT
Bt BsaM dn rev	CGGTACCCGGGGATCCAGCAGCGAGAAAGAAACGAA
Expression in mammalian cells	
BopA for	GAGGAGTGGATGATCAATGTCG
BopA rev	GTTCTTCATGCTGTCTTCAGGC
BopC for	AAGCTTAAGATGCCGAGCATGACC
BopC rev	GGATCCTCATGCGAGTGGGGTGTC
Real-Time PCR	
BopA RT for	TTCGTGCTGTTCGGGCTGA
BopA RT rev	TCGATACTTGAGCTCGCCTGACTT
BopC RT for	TACCGACGATCTCGTCAAAG
BopC RT rev	GCGTTCAAGGAAGTTAAGCC
BopE RT for	TCCTTCGCTTCGCTGAAGATCG [18]
BopE RT rev	ATTCGGCCGGCAAGTCTACG [18]
GAPDH for	CAATGACCCCTTCATTGACC [44]
GAPDH rev	GTTCACACCCATGACGAACATG [44]

### Plasmid transfection and NFκB reporter assay

HEK293T cells were seeded at a density of 1.25x10^5^ cells/well in 24-well tissue culture plates and incubated for 24 hr. For measuring the activation of NFκB by *B. pseudomallei* wildtype (KHW) and mutants, the cells were transfected with 100 ng of pNFκB -SEAP plasmid using jetPRIME DNA & siRNA transfection reagent (Polyplus Transfection). After another 24 hr., the media were replaced with antibiotics-free media. The cells were then infected with mid log-phase cultures of *B. pseudomallei* at required MOI. Following infection, plates were centrifuged at 200 x *g* for 5 min to allow maximum bacteria to cell contact. Two hr. post infection, 250 μg/ml kanamycin was added to kill off extracellular bacteria. Cells without infection were included as control. Supernatant was collected at various time points and SEAP activity was measured. For measuring the activation of NFκB by *B. pseudomallei* T3SS3 effectors, the cells were co-transfected with 100 ng of pNFκB -SEAP plasmid and up to 400 ng of plasmid harbouring *B. pseudomallei* T3SS3 effector gene or 400 ng of empty plasmid using jetPRIME DNA & siRNA transfection reagent. Total amount of DNA transfected were kept constant at 500 ng. After another 24 hr., supernatant was collected and SEAP activity was measured. SEAP activity was measured using Phospha-Light kit (Life Technologies) according to the instructions of the manufacturer. Relative NFκB activation was calculated by averaging the raw luminescence values obtained using the Phospha-Light kit and converting them to fold activation with respect to uninfected cells or cells transfected with empty vector.

### Intracellular bacterial count

HEK293T cells were seeded and infected as described above. Two hr. post infection, cells were washed twice with 1x PBS before addition of fresh culture medium with 250 μg/ml kanamycin. At respective time points, infected cells were washed with 1x PBS and lysed with 0.1% (v/v) Triton X-100. Serial dilutions were performed on the lysates and subsequently plated on TSA agar and incubated at 37°C for 48 hr. Colony counts were used to calculate bacterial loads.

### Cytotoxicity of B. pseudomallei against HEK293T cells

HEK293T cells (1.25 x 10^5^ cells/well) were seeded and grown overnight in a 24 well plate. Cells were infected with the indicated MOI. At 1 hour post infection, kanamycin (250 μg/ml) was added to kill extracellular bacteria. Cytotoxicity was measured at 6 hr. post infection by assaying for lactate dehydrogenase (LDH) release in the cell supernatants using a LDH Cytotoxity Detection Kit (Clontech).

### Multi-nucleated giant cell assay

HEK293T cells were seeded at a density of 2.5 x 10^4^ cells/well in a 24-well tissue culture plate and infected with log-phase bacteria at MOI 10:1. Two hr. post infection, kanamycin was added to kill off extracellular bacteria and at respective time points, cells were washed with 1xPBS and fixed with 100 % methanol (Sigma-Aldrich) for 1 min. Cells were then rinsed with water and air dried before the addition of 20x diluted Giemsa stain (Sigma-Aldrich) for 20 min. After staining, cells were washed with water two times before they were air dried and examined under light microscope for MNGC formation.

### Cloning of full-length bopA, and bopC gene into mammalian expression vector

The pcDNA3.1/V5-His TOPO (pcDNA3.1) TA Expression kit (Life Technologies) was used for cloning of full-length *bopA* for over-expression in mammalian systems. The *bopA* coding sequence including stop codon was included in the primer so that the products were not tagged. Amplified product was cloned into the linearized pcDNA3.1 vector according to manufacturer’s protocol. The *bopC* was cloned into pCMV-FLAG-MAT-Tag-1 Expression Vector (Sigma) according to manufacturer’s instruction. The primers for amplification of *bopA* and *bopC* are listed in Table 3.

### Measurement of B. pseudomallei effector gene expression by real-time PCR

Total RNA was isolated from transfected HEK293T cells 24 hours post transfection using illustra RNAspin Mini Kit (GE Healthcare). cDNA was synthesized using 1 μg of RNA and the First Strand cDNA Synthesis Kit (Thermo Scientific). Transcripts were quantified using iQ Cybr Green Supermix (Bio-Rad) in a Bio-Rad iQ5 machine. The expression of effector gene was normalized to housekeeping control gene *gapdh*. Real-time PCR primers are listed in Table 3.

### Photothermal nanoblade delivery of bacteria

Bacteria for photothermal nanoblade injection were prepared by culturing in low-salt L- broth at pH 5.8 until log-phase and then washed 3X and resuspended in Hanks balanced salt solution (HBSS) at 10^8^–10^9^ cfu/mL. 1–2 μl of the bacterial suspension was loaded into titanium-coated pulled-glass microcapillary pipettes. Photothermal nanoblade delivery was performed essentially as described [24,26]. Briefly, the pulsed laser system used was a Q-switched, frequency-doubled Nd:YAG laser (Minilite I, Continuum) operated at 532 nm wavelength and 6 ns pulsewidth. The laser beam was sent into the fluorescence port of an inverted microscope (AxioObserver, Zeiss) and then through the objective lens (40X, 0.6 NA), to generate a 260 μm-wide laser spot on the sample plane. The optimized laser intensity used for bacterial delivery was 180 mJ/cm^2^. The excitation laser pulse was synchronized with a liquid delivery system (FemtoJet, Eppendorf) using an electronic switch. A pressure of 100–350 hPa was used to deliver 1–2 pl of suspension per pulse. Approximately one bacterium was successfully delivered into a cell every two pulses. Following nanoblade delivery, cells were washed twice with HBSS before the addition of fresh medium with 250 μg/mL kanamycin [24,26].

### Immunoprecipitation

HEK293T cells were first seeded in a 6 well plate at a density of 1 x 10^6^ cells per well and then infected with the required strain the following day. At required time points, cells were lysed with lysis buffer (50 mM Tris pH 7.5, 0.1 mM EGTA, 0.27 M sucrose, 50 mM sodium fluoride, 1 mM sodium orthovanadate, 5 mM sodium pyrophosphate, 1% Triton-100, protease inhibitor cocktail). Protein G sepharose beads (Sigma-Aldrich) were pre-incubated with total TAK1 antibody (kind gift from Dr. Peter Cheung, Nanyang Technological University, Singapore) before the cell lysates were mixed and incubated with the beads for 1 hr. at 4°C with shaking. Beads were then washed twice with lysis buffer and twice with wash buffer (50 mM Tris–HCl pH 7.5, 0.27 M Sucrose, 0.1% 2-mercaptoethanol) before being boiled in SDS-PAGE sample buffer. Samples were subsequently resolved on SDS-PAGE gels and transferred onto nitrocellulose membrane (Pall Life Sciences).

### Western blotting

Cells were lysed with MPer mammalian protein extraction reagent (Thermo Scientific) supplemented with protease cocktail (Thermo Scientific). Proteins were then quantitated using Bradford reagent (Bio-Rad). Samples were boiled in SDS-PAGE sample buffer and 50 μg (per lane) were resolved on an SDS-PAGE gel and transferred onto nitrocellulose membranes (Pall Life Sciences). The membranes were then blocked with 5% BSA at room temperature for 1 hr. and probed with specific antibodies at 4°C overnight followed by secondary antibody anti-rabbit IgG, HRP-linked for 1 hr. at room temperature. Antibodies were obtained from Cell Signaling Technology except the β -actin antibody (Sigma-Aldrich). Blots were developed on film (Pierce Chemical) using ECL plus Western blotting substrate (Thermo Scientific).

### Statistical analysis

NFκB reporter assays were performed in triplicates. Results were presented as mean ± standard deviation. Student’s t-test was used to find the significant differences between the means. The significant differences were reported as *p* < 0.05 (*) and *p* < 0.01 (**).

## Competing interests

The authors declare that they have no competing interests.

## Authors’ contributions

BET, CTF, YHG designed the experiments. BET, YC, IGJC and CTF performed the experiments. BET, YC, CTF, YHG analyzed the results. THW, ES, PYC and MAT set up the photothermal nanoblade experiments. YHG conceived the study and together with CTF and JFM wrote the manuscript. All authors read and approved the final manuscript.
